# Psychiatric nurses’ knowledge of COVID-19 within a patient care context: A qualitative study

**DOI:** 10.4102/sajpsychiatry.v31i0.2344

**Published:** 2025-01-22

**Authors:** Sandisiwe Dyonase, Isabelle Swanepoel, Gian Lippi

**Affiliations:** 1 Department of Psychiatry, Faculty of Health Sciences, University of Pretoria, Pretoria, South Africa

**Keywords:** COVID-19, nurses, knowledge, psychiatric, hospital

## Abstract

**Background:**

Coronavirus disease 2019 (COVID-19) infection caused unparalleled hastening of the transmission of infection worldwide, commonly affecting healthcare workers’ well-being. Nursing staff spend most hours caring for patients and are the first contact that patients utilise when reporting symptoms or receiving treatment.

**Aim:**

This study aims to evaluate the knowledge of COVID-19 among psychiatric nurses at a tertiary psychiatric hospital.

**Setting:**

Weskoppies Hospital, Gauteng, South Africa.

**Methods:**

We conducted a qualitative study comprising 14 semi-structured interviews with nurses working at Weskoppies Hospital in South Africa. We used open-ended questions to facilitate the discussion and provide some structure for the interview while still allowing the participants to elaborate freely. The recordings were later transcribed into text.

**Results:**

Twenty nurses working full time at the hospital, were recruited for the study. The nurses’ knowledge about COVID-19 was summarised into five major themes, each with subthemes: signs and symptoms of COVID-19, risk of contracting the virus, the spread of COVID-19, prevention, and complications. In this study, the majority of participants had relatively good knowledge regarding COVID-19.

**Conclusion:**

The majority of nurses at the hospital had adequate knowledge about COVID-19 but limited knowledge about the mode of transmission of the infection. Consistently improving healthcare workers’ knowledge about infection control measures through training, supplying information and identifying areas for improvement can ultimately enhance patient care and outcomes.

**Contribution:**

This study sheds light on the value of nurses’ understanding of COVID-19, particularly in a psychiatric setting.

## Introduction

A wide range of healthcare workers are involved in caring for patients with coronavirus disease 2019 (COVID-19). Nurses’ knowledge about COVID-19 is crucial as they are in close contact with COVID-19 cases. Therefore, their knowledge might help in preventing transmission. Coronavirus disease 2019 was first identified in the Wuhan region of China in December 2019. It was initially identified as a type of severe acute respiratory distress caused by a coronavirus (SARS-CoV-2), but has now been officially named COVID-19.^1^ The disease presents with the following symptoms: fever, dry cough, tiredness, body aches, sore throat, diarrhoea, conjunctivitis, headache, loss of taste, loss of smell, skin rash and discolouration of fingers and toes. The most serious symptoms include shortness of breath and chest pain. Around 20% of patients may experience severe symptoms requiring oxygen therapy or other inpatient interventions, but only 5% of these will require hospitalisation in an intensive care unit.^2^

The disease was first detected in China and spread to other Asian countries such as South Korea, Malaysia, Philippines and Japan before the epicentre moved to Europe. In Europe, the United Kingdom (UK), Russia, Italy, France and Spain were the focus of the pandemic with higher numbers of infections and deaths compared to those recorded in Asia.^3^ At the time of writing, the countries with the highest number of infections were the United States, Brazil, India, Russia and the UK. South Africa’s first case of COVID-19 was recorded on 11 March 2020. COVID-19 cases started increasing significantly from mid-March 2020 reaching the highest daily recorded number of cases in its first wave. South Africa had the highest number of cases in Africa and was ranked 17th globally.^4,5^ COVID-19 prevention strategies include social distancing, wearing face masks, washing and sanitising hands with alcohol-based solutions and national lockdowns to minimise the spread. Many treatment modalities to prevent severe illness were tried and widely reported. The current attention and focus is on vaccinations.^2,6^

Healthcare workers remain the population at highest risk of contracting and spreading the disease because of the nature of their work and their proximity to ill patients. Inadequate knowledge and incorrect attitudes among healthcare workers can directly influence practices and lead to delayed diagnosis, poor infection control and spread of the disease. With guidelines continuously adapting to current knowledge, it became apparent that healthcare workers needed to keep up-to-date with knowledge and practices. Many studies have thus sought to evaluate the knowledge, attitudes and practices of healthcare workers in relation to COVID-19. Few studies have assessed the knowledge, attitudes and practices of healthcare workers in South Africa and none, to our knowledge, have been conducted in a psychiatric setting. Psychiatric hospitals face unique challenges in managing COVID-19, including difficulty adhering to infection control measures because of the complexity of the patients, as well as resource and infrastructure challenges. The lack of knowledge about COVID-19 might affect the spread of the virus, and it is crucial to determine the extent of nurses’ knowledge about the pandemic.

Weskoppies Hospital is a tertiary academic psychiatric hospital involved in the care of inpatients and outpatients. Nursing staff spend most of their hours caring for patients, are in closest contact with patients and are often the first contact that patients utilise when reporting symptoms. Occupational health and safety staff in the facility conducted training with different healthcare workers when the COVID-19 pandemic was announced in March 2020. Training was provided regarding the donning and doffing of personal protective equipment, common symptoms, modes of transmission, testing, screening, notification and preventive measures, among others.

The primary aim of this study was to gain a clearer understanding of nurses’ knowledge in relation to COVID-19 in a psychiatric institution using semi-structured interviews. Participants who met the criteria were interviewed. Thus, the study contributes to a better understanding of how much this group of nurses knows about the COVID-19 pandemic.

## Methods

### Design

This is a qualitative case study, making use of individual interviews of nurses working at a Weskoppies Hospital in Gauteng.

### Setting

This qualitative study was conducted at Weskoppies Hospital in Gauteng, South Africa.

### Study population and sampling

The study population included 20 full-time nursing staff working in different wards of the hospital and the outpatient department. Participants were sampled by purposive sampling; in other words, every nurse who was willing to participate and who met the criteria of the study were included in the study within the time-frame available to the principal investigator for data collection.

### Interviews

Data were collected during semi-structured interviews, from November 2021 to February 2022. The final interview guide consisted of six open-ended questions or statements that explored the knowledge of COVID-19: (1) What are the common signs and symptoms of COVID-19? (2) Briefly describe who is at risk for infection with SARS-CoV-2, the virus that causes COVID-19. (3) Which modes of transmission of SARS-CoV-2 do you know, and which bodily fluids can spread the infection? (4) Describe the testing, screening and notification involved. (5) What treatment options do you know of? (6) What are the complications of the infection?

### Inclusion and exclusion criteria

The study included full-time professional and assistant nurses between the ages of 34 and 55 years, working in different wards and the outpatient department, and being employed by the hospital.

Individuals who were unwilling to participate and those who did not meet the inclusion criteria were excluded from the study.

### Data collection

Interviews were conducted in English, IsiXhosa or IsiZulu. The principal researcher conducted the semi-structured interviews, which were approximately 15 min – 20 min in duration. A range of open-ended questions were prepared and used as a guide for each interview. Basic demographic data were captured in a data-collection sheet. The questions were designed to stimulate discussion, while adding structure. The questions were developed based on the researcher’s perceptions of what would get the interview started. The participants were encouraged to elaborate freely on every question. The interviewer took field notes during interviews. Each interview was audio recorded and transcribed for further analysis. Data from 20 participants were included. All participants gave written consent. Confidentiality and safety guidelines were implemented at all times. Numbers to protect identities were used to ensure anonymity. The interviews were conducted in private, well-ventilated consultation rooms, and COVID-19 protocols were observed at all times.

### Data analysis

The transcriptions and field notes were analysed using thematic content analysis. This was performed by reviewing available data till the researcher was familiar with its content, ‘coding’ the data (i.e. identifying the important features of each piece of information gathered with a distinct ‘label’) and grouping common codes together. These groups, or categories, were then reviewed and analysed, until common themes and sub-themes were identified among the data: these were further refined in such a way that they can be more easily linked to the study.^7^ The most common or recurrent themes that emerged from the data were given corresponding codes. After 13 interviews, recurrent themes started to emerge, indicating data saturation.

### Ethical considerations

Ethical clearance was obtained from the University of Pretoria, Faculty of Health Sciences’ Research Ethics Committee with reference no: 394/2021. Written informed consent was obtained from the participants. All the information provided, including personal identifiers were treated confidentially.

## Results

Participants ranged in age from 34 to 55 years. Of the 20 nursing staff, 11 were professional nurses, 7 staff nurses and 2 operational managers. As shown in Table 1, there were more women than men. Nursing experience ranged from 7 to 21 years, and they were all working at the hospital at the time of data collection. There was a noticeable difference between participants’ responses for the mode of transmission and prevention domains according to the seniority of participants, that is, operational managers and professional nurses showed higher knowledge than enrolled nurses and assistant nurses in those two domains. Demographic details are summarised in Table 1.

**TABLE 1:** Demographic data of participants.

Demographic	Number
**Gender**	
Male	5
Female	15
**Age (years)**	
34–39	10
40–49	06
50–59	04
**Nursing category**	
Operational manager(s)	2
Professional nurses	11
Enrolled nursing assistant(s)	7
**Years of service**	
7–10	6
11–19	8
20 or more	6

## Themes

From the interviews, we identified five major themes, each with subthemes.

### Theme 1: Nurses’ knowledge of signs and symptoms of COVID-19

#### Subtheme 1.1: Cough, headache, blocked nose, general body pains, loss of taste and appetite

The participants knew the general signs and symptoms of COVID-19. They were aware of this because Occupational Health and Safety staff in the facility trained the different healthcare workers when the COVID-19 pandemic was announced in March 2020:

‘[*Y*]es I know the symptoms and patients usually experience: headaches, cough, fever, loss of taste, chest pains, sore throat.’ (P13, 44-year-old, male)

One of the participants shared her own personal experience:

‘[*A*]t first, well what I have experienced personally, I was having this – my body pains, and then it felt like I was tired and then headache, a very terrible one. I also had a blocked nose, fever and after some time I could not taste my food. Well those are the ones that I experienced …’ (P7, 38-year-old, female)

### Theme 2: Knowledge of risk of contracting COVID-19

#### Subtheme 2.1: Everyone is at risk of contracting COVID-19

Eleven out of 20 participants felt that everybody is at risk of contracting COVID-19. At the time when data were collected, South Africa was in the fourth wave of the pandemic and many people had been affected:

‘I think everybody is at risk, especially if you are exposed to somebody who is infected. You are at a high risk of contracting the virus if you come across an infected person especially if you were exposed to their saliva.’ (P9, 34-year-old, male)

#### Subtheme 2.2: Elderly population with comorbidities

Four out of 20 participants reported that the elderly were the most vulnerable to contracting the virus:

‘This was because more mortalities were recorded amongst the elderly population, especially in patients with comorbidities, during the first wave of the pandemic.’ (P18, 36-year-old, female)

#### Subtheme 2.3: Health workers

Five participants thought that healthcare workers were more prone to contracting the COVID-19 virus as they spend most of their time looking after patients and are in close proximity to patients:

‘It’s us, the healthcare professionals. More especially those that are working in medical wards.’ (P1, 36-year-old, male)

### Theme 3: Mode of transmission of COVID-19

#### Subtheme 3.1: Being in close contact with a COVID-19 positive person

Six out of 20 participants reported that being in close contact with an infected person increased the risk of contracting COVID-19.

#### Subtheme 3.2: COVID-19 spreads through contaminated droplets

Three participants described that COVID-19 was spread through contaminated droplets released when an infected person sneezes or coughs without a mask, especially when the room is not well-ventilated. They further explained that contaminated droplets may settle on surfaces, and if a person touches the contaminated surface and then touches their mouth or eyes, they have an increased risk of contracting the virus.

‘I think the other way to transmit it would be via droplets, like when a person is coughing or sneezing but not wearing a mask and maybe seated very close without opening windows. But sometimes one can touch a contaminated surface with those droplets but they don’t wash hands or sanitise then they get infected.’ (P5, 35-year-old, female)

### Theme 4: Preventing COVID-19

#### Subtheme 4.1: Social distancing, washing and sanitising the hands

Most of the nurses agreed that maintaining hand hygiene, covering the nose and mouth while coughing, and social distancing could help to prevent COVID-19 transmission.

#### Subtheme 4.2: Wearing of personal protective equipment

Although most respondents have had infection prevention and control training from the Occupational and Health and Safety Department in the hospital, the novelty of the COVID-19 pandemic necessitated that extra precautions be taken while caring for patients in the hospital. Constant usage of personal protective equipment (PPE) was a reminder that COVID-19 was novel, and that relaxing self-protection measures was risky to their health:

‘COVID-19 is very contagious and as a result you have to minimise your exposure as a HCW as well as wear PPE all the time.’ (P3, 39-year-old, female)

‘COVID-19 is different from previous illnesses. The difference includes wearing of PPE all the time and full PPE when attending to positive patients.’ (P11, 35-year-old, female)

#### Subtheme 4.3: Avoiding overcrowded spaces

Most participants shared a similar knowledge about avoiding overcrowded spaces, and they entered public spaces that were well-ventilated. They further explained that when in overcrowded spaces, the space between people is reduced and therefore there is no social distancing and the infection spreads more easily among people:

‘Stay at least six feet or 2 meters away from people. Avoid crowds and large gatherings.’ (P4, 42-year-old, male)

### Theme 5: Complications of COVID-19

#### Subtheme 5.1: Death

Most participants were knowledgeable about complications of COVID-19. Eighteen of the 20 participants reported death as a complication of COVID-19:

‘COVID-19 pandemic has caused a significant number of deaths as it was reported in the news worldwide and the actual number of deaths could have been higher due to underreporting and varying definitions of COVID-19- related deaths.’ (P9, 37-year-old, female)

#### Subtheme 5.2: Long-term respiratory problems

Participants reported that older adults and people with certain health conditions have a higher risk of severe complications if they are infected with the virus:

‘COVID-19 has a lot of complications but commonly people continue having breathing problems, I know of a relative who is now on continuous oxygen support.’ (P14, 43-year-old, male)

## Discussion

COVID-19 was a global topic of discussion among healthcare workers and patients during the height of the pandemic. Managing such a crisis requires knowing about how information is managed to help frontline healthcare workers in times of public health crises. Several studies have described the knowledge, attitudes and practices of healthcare workers in relation to COVID-19 all over the globe, but none, to our knowledge, have been conducted in a psychiatric setting. Psychiatric hospitals face unique challenges in managing COVID-19, including challenging behaviours and psychiatric settings often involving shared living spaces, increasing the risk of transmission. Nurses are expected to know how to manage those challenges while maintaining infection control.

In this study, we assessed nurses’ knowledge of COVID-19 when managing mentally ill patients. Most participants had relatively good knowledge regarding COVID-19, unlike what was found in a study conducted in Iran and Saudi Arabia which highlighted that 42.85% of nurses had excellent awareness about the virus and that more than half of respondents had a positive attitude towards providing care to COVID-19 patients.^8^ A self-administered questionnaire was used to assess knowledge of COVID-19 in Nigeria, using a cross-sectional descriptive method. They concluded that community healthcare workers were grossly underprepared for providing health education on COVID-19, because of their poor level of knowledge.^9^

Moodley et al. used a World Health Organization questionnaire to assess the knowledge, attitudes and practices of healthcare workers in four provinces of South Africa. They found that 67% of healthcare workers scored 6 or more out of 10 on the knowledge items.^10^ In Ghana, healthcare workers had encouraging willingness to handle COVID-19; however, professionals had limited knowledge of the pandemic.^11^ In our study, nurses had good knowledge of the pandemic. The difference between our findings and those of other studies might be because of the fact that we conducted our study after the worst of the pandemic had passed, and we only interviewed nurses who had already received training from the Occupational Health and Safety Department. It is also possible that the seriousness of the global pandemic, in addition to daily updates from public health agencies in respective countries, would have prompted the need to learn and acquire knowledge on COVID-19.

In our study, participants’ knowledge about the mode of transmission of COVID-19 was poor, despite them having received training. This is unfortunate because the surge of COVID-19 was globally devastating, and healthcare authorities provided many resources to educate healthcare workers and improve their knowledge of COVID-19. Knowing that people working in hospitals are at a higher risk of secondary infection or spreading the virus to colleagues, family and friends, nurses should have knowledge of the disease and infection control measures to prevent spread. Empirical data suggest that COVID-19 was challenging to nurses because of the novelty of the disease.

All of the nurses in our study agreed that handwashing and other preventive measures play an important role in reducing infection risk. A similar positive attitude towards most preventive measures was earlier reported in Egypt and India, but the latter noted some reluctance in following some recommendations such as the use of face masks.^12^ In another study conducted in China, most healthcare workers followed health recommendations and less than 4% went to crowded places or went outside without a facemask. Chinese healthcare workers were also optimistic about the success of their COVID-19 control programme.^1,13^

In our study, 96% of participants considered self-isolation essential and effective, and they avoided places with confirmed COVID-19 cases. Similar findings were reported in Egypt and Nigeria,^9,14,15^ where fewer cases were also reported. Nurses in our study were knowledgeable about preventive practices in dealing with COVID-19. A previous study among healthcare workers on MERS-CoV in Saudi Arabia and a recent study about COVID-19 in India among students and health care workers also reported that nurses were able to practise in their full clinical capacity and use preventive measures because of constant exposure and previous outbreak experience similar to COVID-19.^8,15,16^

Health workers in China had varied practices and levels of knowledge because of factors such as job category, work experience, working hours and educational attainment.^1^ Transmission of this virus is associated with overcrowding, absence of isolation facilities and environmental contamination. Transmission would be further exacerbated if healthcare workers had inadequate knowledge about the pandemic.

### Strengths and limitations

The strength of this study is that it is the first study to our knowledge to evaluate nurses’ knowledge about COVID-19 in a psychiatric hospital. Limitations include that this is a single centre study, which limits the generalisability of the findings. The study also had a small sample size. The semi-structured interviews conducted were retrospective in nature, and thus potentially prone to recall bias.

Despite the limitations, our findings provide valuable information regarding nurses’ knowledge about COVID-19 for when they are managing patients, especially in a psychiatric hospital.

## Conclusion

Most of the nurses working at the hospital had adequate knowledge of COVID-19 but limited knowledge on the mode of transmission of the infection. Consistently improving healthcare workers’ knowledge through training, supplying information and identifying areas for improvement can ultimately enhance patient care and outcomes.
